# A Study on Optometrists’ Knowledge, Awareness, and Management of Traumatic Brain Injury-Related Visual Disorders in Saudi Arabia

**DOI:** 10.3390/healthcare13131609

**Published:** 2025-07-04

**Authors:** Nawaf M. Almutairi, Abdulaziz Alharbi, Abdulelah Alharbi, Mohammed M. Alnawmasi

**Affiliations:** Department of Optometry, College of Applied Medical Sciences, Qassim University, Buraydah 51452, Saudi Arabia; 391108604@qu.edu.sa (A.A.); 391108298@qu.edu.sa (A.A.); m.alnawmasi@qu.edu.sa (M.M.A.)

**Keywords:** traumatic brain injury, visual dysfunction, optometry, vision therapy, clinical awareness, management

## Abstract

**Background:** Traumatic brain injury frequently leads to visual dysfunction, affecting up to 75% of patients. These visual issues, if unrecognized, can significantly impair daily functioning. Optometrists are well-positioned to identify and manage such conditions, yet their level of preparedness is not well understood. **Objective**: This study aimed to assess optometrists’ knowledge, awareness, and management practices regarding TBI-related visual disorders in Saudi Arabia. **Methods:** A cross-sectional survey was distributed online to 411 licensed optometrists in Saudi Arabia. The 16-item questionnaire assessed demographics, awareness, confidence, knowledge, and management practices related to TBI-associated visual disorders. **Results:** Only 26.3% of the respondents reported receiving formal education on TBI-related visual disorders. While most recognized common symptoms, such as blurred vision and light sensitivity, comprehensive knowledge of complex visual disorders was limited. A majority (82.5%) recommended referral to other healthcare providers; however, only 16.8% demonstrated high management competency, and 31.5% fell into the low-competency category. Referral patterns and clinical decision-making were significantly associated with experience and formal training. **Conclusion:** The findings reveal notable gaps in optometrists’ knowledge and preparedness to manage TBI-related visual dysfunctions. Structured educational initiatives and standardized clinical protocols are essential to improve optometric care for individuals with TBI.

## 1. Introduction

Traumatic brain injury (TBI) occurs when an external force disrupts normal brain function, commonly due to falls, vehicular accidents, or sports-related injuries [1,2,3,4]. According to the Centers for Disease Control and Prevention (CDC), approximately 2.8 million TBI-related emergency visits, hospitalizations, and deaths occurred in 2013 in the United States alone [1]. These figures underestimate the true incidence, excluding outpatient cases and unreported injuries.

TBI severity is classified as mild, moderate, or severe based on a neurological assessment, often using the Glasgow Coma Scale (GCS) [2]. The mechanisms of injury include direct impact, shear strain, and rotational acceleration, which can cause a cascade of physiological disturbances such as axonal injury [3], altered neurotransmission, and inflammation [4]. These processes may lead to widespread functional impairments, including deficits in memory, short- and long-term cognition, executive functioning, and behavior [5,6].

Given that over half of the brain is dedicated to visual processing, it is unsurprising that visual symptoms occur in up to 75% of patients with TBI [7,8]. These dysfunctions may include oculomotor impairments, such as convergence [9,10], accommodative [11,12,13,14,15,16], and saccadic dysfunction [17]. The non-oculomotor symptoms include light sensitivity, motion sensitivity, visual field defects, and visual processing difficulties [8]. These visual impairments can significantly affect activities of daily living, including reading, driving, and occupational performance [11,18].

Rehabilitation for TBI is inherently multidisciplinary. Occupational, physical, and speech therapists address cognitive, motor, and communicative deficits [19]. Within this team, optometrists play a critical role in identifying and managing TBI-related visual dysfunction. Interventions such as neurorehabilitation have demonstrated efficacy in alleviating visual symptoms and improving quality of life [20]. For example, optometric vision therapy for accommodative dysfunctions has a long-standing scientific foundation and demonstrates high success rates in both children and adults [21,22,23,24,25].

Despite their crucial role [26], little is known about optometrists’ preparedness to manage TBI-related visual conditions, especially in regions where optometric neurorehabilitation is still emerging. In Saudi Arabia, no data exist on optometrists’ knowledge, awareness, and clinical practices in this domain. Therefore, this study aimed to evaluate optometrists’ knowledge, awareness, and management practices regarding TBI-related visual disorders in Saudi Arabia. Specifically, it seeks to (1) assess the current management strategies used by optometrists and (2) determine the extent of their knowledge and awareness related to concussion-associated visual dysfunctions.

## 2. Methods

### 2.1. Study Design and Participants

This study employed a cross-sectional survey design to evaluate the awareness, knowledge, and management practices of optometrists regarding TBI-related visual disorders in Saudi Arabia. The target population included licensed optometrists practicing across different healthcare and clinical settings in Saudi Arabia. A total of 411 optometrists completed the online questionnaire. The study adhered to the guidelines outlined in the Declaration of Helsinki. Participation was voluntary, and informed consent was obtained electronically before survey completion. All data were collected anonymously and treated with strict confidentiality in accordance with institutional guidelines.

### 2.2. Survey Instrument

A 16-item structured questionnaire was developed, consisting of multiple-choice, Likert scale, and multi-select questions, organized into four major domains:

1. Demographics: age, gender, years of experience, and education level.
2. Awareness: frequency of encountering TBI cases, whether they inquire about concussion history, and comfort in managing such cases.
3. Knowledge: formal training, symptom recognition, and knowledge of visual disorders.
4. Management: Management was assessed by asking the participants to identify the evidence-based strategies they are familiar with for managing TBI-related visual dysfunctions. These included common interventions such as vision therapy, prism lenses, tinted lenses, and referral to specialists. The goal was to evaluate the general awareness of possible clinical approaches, not to determine the appropriateness of intervention in individual cases.

The content validity of the survey was established by four clinical experts in optometry and brain injury rehabilitation. Minor wording revisions were made following pilot testing with 10 optometrists; see Appendix A.

### 2.3. Scoring System

Four scoring systems were utilized to evaluate the participants’ awareness, knowledge, and confidence in diagnosis and management related to TBI-associated visual disorders.

*Awareness Score*: Awareness was measured using three survey items: the frequency of encountering patients with TBI-related visual disorders, whether the respondents routinely inquired about a history of concussion or TBI, and whether they felt comfortable managing such cases. The responses were scored as follows: frequency of patient encounters (never = 0, rarely = 1, occasionally = 2, frequently = 3), routine inquiry about TBI history (no = 0, yes = 1), and comfort in managing TBI cases (no = 0, yes = 1). The total awareness score ranged from 0 to 5 and was categorized as low (0–2), moderate (3–4), or high (5) awareness.

*Knowledge Score*: Knowledge was evaluated based on correct responses to three specific questionnaire items related to visual symptoms and disorders commonly associated with TBI. One point was assigned for each correct response. The cumulative score was then categorized into low knowledge (0–1), moderate knowledge (2), and high knowledge (3).

*Confidence Score*: Confidence in diagnosing TBI-related visual disorders was assessed using a single 10-point Likert scale item. The participants were asked to rate their confidence on a scale from 1 (not confident) to 10 (very confident). The responses were grouped into three categories: low confidence (1–4), moderate confidence (5–7), and high confidence (8–10).

*Management Score*: To quantify management competency, a management score was calculated by awarding 1 point for each evidence-based strategy selected, resulting in a total possible score ranging from 0 to 4. Based on the cumulative score, the participants were categorized into three levels of management competency: low (0–1 points), moderate (2–3 points), and high (4 points). This scoring system aimed to evaluate the breadth of the participants’ familiarity with appropriate management options for TBI-related visual disorders.

### 2.4. Data Collection

The data were collected using Google Forms. The survey was distributed electronically via professional groups and email. Participation was voluntary and anonymous.

### 2.5. Statistical Analysis

The data were analyzed using SPSS software (version 25). Descriptive statistics (frequencies, percentages) were calculated for all variables. Chi-square tests were used to examine the associations between the demographic characteristics and the knowledge/confidence categories. A significance level of *p* < 0.05 was considered statistically significant.

## 3. Results

### 3.1. Subjects’ Profiles

A total of 411 optometrists participated in the study. Most respondents were aged 25–34 years (71.9%), followed by those aged 35–44 years (10.7%). Smaller proportions were aged under 25 years (6.6%), 45–54 years (2.9%), and 55 years or older (1.1%). The sample was fairly balanced by gender, with a slight male predominance; see Table 1. Regarding clinical experience, 35.6% reported 2–5 years of professional experience, 24.5% had less than 2 years, 19.5% had 5–10 years, and 13.6% reported more than 10 years of experience in optometric practice. With respect to educational qualifications, the majority of respondents held a bachelor’s degree (80.8%), followed by a master’s degree (15.8%) and a doctorate degree (2.9%); see Table 2.

### 3.2. Awareness

The analysis of the awareness levels among the 411 participating optometrists revealed that a majority demonstrated low awareness regarding TBI-related visual disorders. In total, 61.7% (*n* = 272) of the respondents fell into the low-awareness category, while 30.4% (*n* = 134) were classified as having moderate awareness. Only 7.9% (*n* = 35) of the optometrists exhibited high awareness.

### 3.3. Knowledge of TBI

The level of knowledge about visual disorders did not significantly differ by age (*p* = 0.37), though the younger participants aged 25–34 years were the most represented across all knowledge levels, making up 95.2% of those with low knowledge and 74.0% of those with high knowledge. Similarly, the gender differences were not statistically significant (*p* = 0.07), but males were more prevalent in the low-knowledge group (71.4%) compared to the high-knowledge group (54.5%)

Experience was significantly associated with knowledge (*p* = 0.002). The proportion of those with high knowledge was highest among those with 2–5 years (32.1%) and 5–10 years of experience (22.7%). In contrast, 57.1% of those with low knowledge had only 2–5 years of experience, and only 4.8% had more than 10 years of experience. Education level was not significantly associated with knowledge (*p* = 0.45), though individuals with a doctorate degree were more likely to have high knowledge (4.2%) compared to those with lower degrees; see Table 2 and Table 3.

### 3.4. Differences Based on Formal Education in Concussion Management

To explore the impact of formal education on clinical practice, a Chi-square test of independence was conducted to assess the association between having received formal education/training on concussion-related visual disorders and whether the optometrists routinely asked patients about a history of concussion or TBI. The results showed a significant association between these variables: χ^2^(4, *n* = 411) = 449.86, *p* < 0.001. The optometrists who reported receiving formal training were considerably more likely to inquire about TBI history during clinical assessments.

The proportion of optometrists who reported encountering patients with concussion or traumatic brain injury (TBI) either occasionally or frequently was similarly low across groups—12.5% among those without formal training and 13.0% among those with training—indicating that formal education does not necessarily increase clinical exposure. However, the trained optometrists demonstrated higher levels of clinical engagement in other domains. Confidence in diagnosing TBI-related visual disorders was also notably greater among those who had received training, with 74.9% reporting moderate to high confidence levels (scores 6–10), in contrast to 47.1% in the untrained group. Furthermore, the importance of concussion knowledge to optometric practice was rated as high (scores 8–10) by 76.9% of the trained respondents, compared to 73.0% of those without training; see Table 4.

### 3.5. Confidence in Diagnosis

Confidence in diagnosing concussion/TBI significantly varied across the age groups (*p* = 0.009). The majority of the participants in all confidence levels were aged 25–34 years, comprising 85.6% of those with low confidence, 72.9% of those with moderate confidence, and 69.5% of those with high confidence. Those aged 35–44 years were more likely to report high confidence (20.3%) compared to their younger counterparts, while very few participants over 45 years reported high confidence. Gender also showed a significant association with confidence (*p* < 0.001). Males accounted for 66.8% of those with moderate confidence and 62.7% of those with high confidence, whereas females were more frequently found in the low-confidence category (56.2%).

Professional experience significantly influenced confidence levels (*p* < 0.001). Among those with high confidence, 30.5% had more than 10 years of experience, compared to only 4.6% in the low-confidence group. Conversely, less than two years of experience was most common among those with low confidence (28.8%) and least common in the high-confidence group (16.9%). Education was not significantly associated with confidence (*p* = 0.09), though the participants with doctorate degrees were more frequently in the high-confidence group (8.5%) than in the moderate- (3.0%) or low- (2.0%) confidence groups; see Table 5.

The scores of confidence in diagnosing concussion/TBI generally increased with experience and education level, with those having more than 10 years of experience and doctorate degrees showing higher median confidence scores. Males also displayed slightly higher confidence levels than females.

### 3.6. Management

The participants were asked to select the evidence-based management strategies they would personally recommend for patients with concussion/TBI-related visual disorders. Among the 411 respondents, the majority selected referral to a neurologist or other specialist (82.5%, *n* = 339), followed by vision therapy (61.3%, *n* = 252), prism lenses (49.4%, *n* = 203), and tinted lenses (28.5%, *n* = 117). A small number of the participants (0.7%, *n* = 3) selected “none of the above,” and several provided individualized responses under “other,” such as “plenty of rest,” “orthoptic report,” or “depends on the case.”

To assess the overall management competency of the respondents, a management score was calculated based on the number of evidence-based strategies selected, with a maximum possible score of 4. The scores were then categorized into low (0–1), moderate (2–3), and high (4) levels of management competency. The results are shown in Table 6.

These findings indicate that while most participants were able to identify at least one or two appropriate strategies, a relatively small proportion demonstrated comprehensive knowledge of the full range of optometric management options for TBI-related visual disorders. This suggests a need for improved training to support more consistent and independent clinical decision-making.

### 3.7. Referral Patterns for Concussion-Related Visual Disorders

A Chi-square test of independence was conducted to assess the relationship between receiving formal education or training on concussion-related visual disorders and the frequency of referring patients with concussion/TBI-related visual conditions to other healthcare providers. The results revealed a statistically significant association between these variables: χ^2^(8, *n* = 411), *p* < 0.001. Among the optometrists with formal education (*n* = 108), 48.1% reported always referring patients and 37.0% reported doing so sometimes, while smaller proportions reported rarely (8.3%) or never (6.5%) referring. A similar trend was seen among those without training (*n* = 303), with 48.8% always, 24.1% sometimes, 14.2% rarely, and 12.9% never referring patients.

Although the trained and untrained respondents had comparable rates of “always” referring, those with training showed lower rates of non-referral (never/rarely) and a higher tendency toward occasional referral (sometimes). This pattern suggests that formal education may contribute to more consistent and nuanced referral practices, potentially reflecting an increased awareness of concussion-related visual dysfunction and collaborative management pathways.

The respondents were allowed to select multiple healthcare providers to whom they refer patients with concussion/TBI-related visual disorders. The most common referral destinations were ophthalmologists (77.1%) and neurologists (58.7%), followed by occupational therapists (27.7%), other optometrists (16.6%), physical therapists (16.1%), and speech/language therapists (9.3%). These patterns indicate that optometrists frequently engage in interdisciplinary referrals, with a preference for medical and rehabilitative specialists.

## 4. Discussion

The findings of this study highlight significant gaps in the awareness, knowledge, and management practices of optometrists in Saudi Arabia concerning TBI-related visual disorders. These gaps are notable given the high prevalence of visual disturbances among TBI patients and the critical role optometrists play in their diagnosis and treatment.

The survey results reveal that a large proportion of the optometrists infrequently encounter TBI patients, with only 1.7% frequently seeing such cases. Additionally, only about half of the respondents consistently inquire about the history of TBI, and a mere 26.3% have received formal education on TBI-related visual disorders. This aligns with findings from Lacroix et al. (2018), who noted similar training deficiencies among Canadian optometrists [26]. This lack of structured education likely contributes to the moderate confidence levels in diagnosing TBI-related visual disorders and the under-screening of patients in clinical settings. However, the low reported frequency of TBI patients presenting to optometric clinics may reflect broader trends in patient pathways, where concussion-related symptoms are often initially assessed in emergency departments, general medical practices, or neurology clinics. This referral pattern may contribute to an under-recognition of visual symptoms by optometrists. Nonetheless, optometrists remain in a pivotal position to identify unrecognized visual dysfunctions during routine eye examinations, particularly in populations with a history of head trauma [27]. This aligns with studies demonstrating that convergence insufficiency, accommodative infacility, and oculomotor anomalies often remain under-recognized without specific probing during the clinical exam [11,15].

Despite the recognition of common visual symptoms, such as blurred vision and light sensitivity, our results showed variability in comprehensive knowledge, especially regarding less obvious symptoms and disorders like convergence insufficiency and accommodative anomalies. This is consistent with prior studies reporting knowledge gaps with regard to the visual dysfunctions associated with TBI [28].

The statistical analysis revealed significant associations between professional experience and diagnostic confidence and between experience and knowledge level. These findings support the idea that hands-on clinical exposure enhances both awareness and competence in managing TBI-related visual disorders.

Although the optometrists demonstrated some understanding of the appropriate management strategies—such as vision therapy and the use of prism lenses—only 16.8% were categorized as having high management scores. High referral rates to neurologists and ophthalmologists, as shown in our data, may reflect limited confidence or uncertainty about the scope of optometric management. This observation underscores the importance of enhancing training pathways and clinical decision-making frameworks for optometrists.

While the majority of the respondents indicated some familiarity with management strategies, the data do not clarify whether these approaches are applied appropriately in clinical contexts. Referral decisions, in particular, were not tied to specific symptom profiles or case types. It is important to note that the current practice guidelines suggest that optometrists should triage patients with suspected TBI-related visual disorders based on symptom severity, type of visual dysfunction, and multidisciplinary needs [11]. Comprehensive assessment and referral to neuro-optometrists, occupational therapists, neurologists, or vision rehabilitation services may be warranted depending on the case complexity.

It is also important to clarify that the inclusion of interventions such as vision therapy or prism lenses in this study was not intended to imply they are universally appropriate treatments for all TBI visual disorder cases. Their use should be guided by a comprehensive clinical evaluation and tailored to the specific functional deficits presented by the patient. This reinforces the need for standardized guidelines and education to support evidence-based, individualized care.

Our findings also revealed that the optometrists who had received formal education were significantly more likely to inquire about TBI history and refer patients appropriately. This suggests that training plays a direct role in improving clinical vigilance and collaborative care behaviors. Similar conclusions were drawn by Haarbauer-Krupa et al. (2017), who emphasized structured training as a driver of interdisciplinary collaboration [29].

Interestingly, the knowledge and confidence levels were not significantly associated with gender or education level, although a slightly higher confidence level was observed among males and those with doctoral degrees. This suggests that, beyond academic qualifications, clinical experience and ongoing professional development are likely more influential factors.

In sum, these results support the growing body of evidence advocating for the integration of TBI-focused education within optometric curricula and continuing education programs. Ensuring that optometrists are adequately equipped to recognize, manage, and refer TBI-related visual disorders is critical for patient outcomes, especially given the complex and multifaceted nature of these impairments.

Future research should explore the development and evaluation of targeted educational interventions and practice guidelines for TBI-related visual dysfunctions within the optometric community.

## 5. Limitations

1. The data were obtained from optometrists working in various settings, including governmental hospitals with abundant facilities and optical shops with potentially fewer resources. This variability may influence the findings, as optometrists in hospitals may encounter more TBI patients and have access to more diagnostic and therapeutic tools.
2. The categorical classification of experience levels prevented an in-depth analysis of the impact of experience on awareness, knowledge, and management practices.
3. Finally, the survey did not include case-based or scenario-driven items to evaluate how optometrists would apply their knowledge in real-world clinical decision-making. This limits the depth of insight into actual management competence. Future studies should incorporate clinical vignettes or simulated cases to assess not only the awareness of management options but also the appropriateness of the decisions made in specific patient contexts. Although the instrument underwent expert review and pilot testing, a formal psychometric validation, including comparisons with established tools, was not conducted.

## 6. Conclusions

This study highlights that while most optometrists in Saudi Arabia infrequently encounter patients with TBI-related visual disorders, there is significant variability in their confidence and knowledge levels regarding the management of these conditions. Only a quarter of the optometrists surveyed have received formal education on TBI-related visual disorders, and just over half routinely inquire about a history of TBI or head injury. Despite this, a substantial proportion recognize the importance of understanding these disorders and recommend appropriate management strategies. These findings suggest a need for enhanced education and training programs to better equip optometrists in Saudi Arabia to effectively manage TBI’s visual consequences.

## Figures and Tables

**Table 1:** Age and gender distribution of the study participants (*n* = 411).

Age (a)	Gender		Total	*n* (%)
	M	F		
**Under 25**	14	15	29	29 (7.1)
**25–34**	178	139	317	317 (77.1)
**35–44**	32	15	47	47 (11.4)
**45–54**	9	4	13	13 (3.2)
**55 or older**	4	1	5	5 (1.2)
**Total**	237	174	411	411 (100.0)

**Table 2:** Knowledge score by experience level.

	Low Knowledge	Moderate Knowledge	High Knowledge	*p*-Value
	(*n* = 21)	(*n* = 82)	(*n* = 308)	
Age				
Under 25	1 (4.8%)	3 (3.7%)	25 (8.1%)	
25–34	20 (95.2%)	69 (84.1%)	228 (74.0%)	0.37
35–44	0 (0%)	7 (8.5%)	40 (13.0%)	
45–54	0 (0%)	2 (2.4%)	11 (3.6%)	
55 or older	0 (0%)	1 (1.2%)	4 (1.3%)	
Gender				
Female	6 (28.6%)	28 (34.1%)	140 (45.5%)	0.0777
Male	15 (71.4%)	54 (65.9%)	168 (54.5%)	
Experience				
Less than 2 years	5 (23.8%)	14 (17.1%)	89 (28.9%)	0.00286
2–5 years	12 (57.1%)	46 (56.1%)	99 (32.1%)	
5–10 years	3 (14.3%)	13 (15.9%)	70 (22.7%)	
More than 10 years	1 (4.8%)	9 (11.0%)	50 (16.2%)	
Education				
Bachelor’s degree	17 (81.0%)	68 (82.9%)	247 (80.2%)	0.45
Master’s degree	3 (14.3%)	14 (17.1%)	48 (15.6%)	
Doctorate degree	1 (4.8%)	0 (0%)	13 (4.2%)	

**Table 3:** Descriptive statistics of indicators related to knowledge and management.

	Overall
	(*n* = 411)
Encounter patients with concussion/TBI	
Frequently	7 (1.7%)
Never	118 (28.7%)
Occasionally	45 (10.9%)
Rarely	241 (58.6%)
Routinely ask history of concussion/TBI	
No	201 (48.9%)
Yes	210 (51.1%)
Comfortable managing patients with concussion/TBI	
No	193 (47.0%)
Yes	218 (53.0%)
Formal education or training	
No	303 (73.7%)
Yes	108 (26.3%)
Referral frequency to other healthcare providers	
Always	200 (48.7%)
Never	46 (11.2%)
Rarely	52 (12.7%)
Sometimes	113 (27.5%)
Where patients are referred by optometrists (multiple options)	
Ophthalmologist	274 (62.0%)
Neurologist	274 (62.0%)
Another optometrist	55 (12.4%)
Physical therapist	47 (10.6%)
Occupational therapist	74 (16.7%)
Speech/language therapist	43 (9.7%)

**Table 4:** Comparison of key clinical behaviors and perceptions among optometrists with and without formal concussion-related training.

Domain	No Training (*n* = 303)	Formal Training (*n* = 108)
Frequency of encountering TBI patients (occasionally/frequently)	38 (12.5%)	14 (13.0%)
Routinely ask about concussion history	142 (46.9%)	68 (63.0%)
Confidence in diagnosis	143 (47.1%)	81 (74.9%)
Importance of concussion knowledge	221 (73.0%)	83 (76.9%)

**Table 5:** Results of confidence to diagnose concussion/TBI by demographic characteristics along with Chi-square test.

	Low Confidence	Moderate Confidence	High Confidence	*p*-Value
	(*n* = 153)	(*n* = 199)	(*n* = 59)	
Age				
Under 25	10 (6.5%)	16 (8.0%)	3 (5.1%)	
25–34	131 (85.6%)	145 (72.9%)	41 (69.5%)	0.00964
35–44	12 (7.8%)	23 (11.6%)	12 (20.3%)	
45–54	0 (0%)	11 (5.5%)	2 (3.4%)	
55 or older	0 (0%)	4 (2.0%)	1 (1.7%)	
Gender				
Female	86 (56.2%)	66 (33.2%)	22 (37.3%)	<0.001
Male	67 (43.8%)	133 (66.8%)	37 (62.7%)	
Experience				
Less than 2 years	44 (28.8%)	54 (27.1%)	10 (16.9%)	<0.001
2–5 years	72 (47.1%)	69 (34.7%)	16 (27.1%)	
5–10 years	30 (19.6%)	41 (20.6%)	15 (25.4%)	
More than 10 years	7 (4.6%)	35 (17.6%)	18 (30.5%)	
Education				
Bachelor’s degree	122 (79.7%)	167 (83.9%)	43 (72.9%)	0.09
Doctorate degree	3 (2.0%)	6 (3.0%)	5 (8.5%)	
Master’s degree	28 (18.3%)	26 (13.1%)	11 (18.6%)	

**Table 6:** Distribution of optometrists’ management competency levels in handling TBI-related visual disorders, categorized as low, moderate, and high based on the number of evidence-based strategies selected.

Management Competency Level	Number (*n*)	Percentage (%)
Low	139	31.5%
Moderate	198	44.9%
High	74	16.8%

## Data Availability

Data is contained within the article.

## Appendix A

Instructions:

Please answer the following questions to the best of your knowledge and experience. Your responses will be kept confidential and used for research purposes only.

Demographics:

1. What is your age?
  A. Under 25
  B. 25–34
  C. 35–44
  D. 45–54
  E. 55 or older

2. What is your gender?
  A. Male
  B. Female

3. What is your level of experience in optometry?
  A. Less than 2 years
  B. 2–5 years
  C. 5–10 years
  D. More than 10 years

4. What is your highest level of education?
  A. Bachelor’s degree
  B. Master’s degree
  C. Doctorate degree

Awareness:

5. How often do you encounter patients with TBI-related visual disorders in your practice?
  A. Never
  B. Rarely
  C. Occasionally
  D. Frequently

6. Do you routinely ask patients about a history of concussion or head injury?
  A. Yes
  B. No

7. Do you feel comfortable managing patients with concussion-related visual disorders?
  A. Yes
  B. No

8. How confident are you in your ability to diagnose TBI-related visual disorders?

Scale from 1 to 10: 1 being not confident, and 10 being very confident.

Knowledge:

9. Have you received any formal education/training on concussion-related visual disorders?
  A. Yes
  B. No

10. Which visual symptoms are commonly associated with concussions? (Select all that apply)
  A. Blurred vision
  B. Double vision
  C. Light sensitivity
  D. Eye fatigue
  E. Eye movement problems
  F. None of the above

11. Which of the following is a common symptom of post-concussion syndrome?
  A. Dizziness
  B. Nausea
  C. Headache
  D. All of the above
  E. None of the above

12. Which of the following visual disorders are commonly associated with concussion/TBI? (Select all that apply)
  A. Convergence insufficiency
  B. Accommodative anomalies
  C. Eye movement dysfunction
  D. Delayed visual processing
  E. None of the above
  F. All of the above

13. How important do you think it is for optometrists to be knowledgeable about concussion/TBI-related visual disorders?

Scale from 1 to 10: 1 being not confident, and 10 being very confident.

Management:

14. Which of the following management strategies would you recommend for a patient with a concussion-related visual disorder? (Select all that apply)
  A. Vision therapy
  B. Prism lenses
  C. Tinted lenses
  D. Referral to a neurologist or other specialist
  E. None of the above
  F. Other

15. How often do you refer patients with concussion-related visual disorders to other healthcare providers?
  A. Always
  B. Sometimes
  C. Rarely
  D. Never

16. To what healthcare providers often do you refer patients with concussion/TBI-related visual disorders? (Select all that apply)
  A. Ophthalmologist
  B. Another optometrist
  C. Occupational therapist
  D. Physical therapist
  E. speech/language therapist
  F. Neurologist

Conclusion:

Thank you for participating in this study. Your responses will help us better understand optometrists’ knowledge, awareness, and management of traumatic brain injury-related visual disorders.
